# Surgical approach and oncological outcomes in Siewert type II junctional tumors

**DOI:** 10.1093/dote/doag049

**Published:** 2026-05-20

**Authors:** Adam Zeyara, Gino M Kuiper, Stijn Vanstraelen, Jelle P Ruurda, Richard van Hillegersberg

**Affiliations:** Department of Surgery, UMC, Utrecht, The Netherlands; Department of Surgery, Skane University Hospital, Lund, Sweden; Department of Surgery, UMC, Utrecht, The Netherlands; Department of Surgery, UMC, Utrecht, The Netherlands; Department of Surgery, University Hospitals Leuven, Belgium; Department of Surgery, UMC, Utrecht, The Netherlands; Department of Surgery, UMC, Utrecht, The Netherlands

**Keywords:** esophagectomy, extended total gastrectomy, esophagogastric junction cancer

## Abstract

Optimal surgery for Siewert type II tumors remains controversial. We compared outcomes between transthoracic esophagectomy (TTE) and extended total gastrectomy (EG) in a high-volume center. This study included consecutive patients with Siewert type II tumors undergoing curative-intent surgery between 2013 and 2023. Patients treated with TTE or EG were analyzed. Primary outcomes were oncological parameters (lymph node yield, margin length, R0-rate, positive lymph nodes). Secondary outcomes included postoperative morbidity, overall survival (OS), disease-free survival (DFS), and recurrence patterns. Survival was analyzed using Kaplan–Meier and multivariable Cox regression. A total of 136 patients were included (TTE *n* = 71; EG *n* = 65). Baseline characteristics were largely comparable. TTE resulted in a significantly higher median lymph node yield (45 vs. 28, *P* < 0.001) and longer proximal margins (*P* < 0.001). R0 resection rates were similar (94.4% vs. 86.0%, *P* = 0.217), as was the number of positive lymph nodes. Clavien–Dindo ≥3b complications occurred in 38.0% vs. 38.5% (*P* = 1.000). Median OS was 2.9 years in both groups, with no difference in OS (log-rank *P* = 0.611) or DFS (log-rank *P* = 0.530). On multivariable analysis, surgical approach was not associated with OS (aHR 1.08, 95% CI 0.64–1.82), whereas advanced pathological stage (pT3–4) independently predicted worse survival (aHR 2.03, 95% CI 1.18–3.51). Despite differences in lymph node yield and proximal margin length, TTE and EG provide comparable oncological and survival outcomes in Siewert type II tumors. These results were sustained even after multivariate adjustment. These findings support an individualized, multidisciplinary approach to surgical strategy selection.

## INTRODUCTION

Esophagogastric junctional tumors are classified according to the Siewert classification, having both therapeutic and diagnostic implications.^1^ True gastroesophageal junction (Siewert type II) tumors can be treated with either a transthoracic esophagectomy or an extended total gastrectomy, depending on the axial extent of the primary tumor and extent of lymph node involvement.^2^ Both approaches strive to achieve complete resection of the primary lesion and an adequate lymphadenectomy, but controversy persists regarding which approach offers superior surgical and oncological outcomes, as well as better survival and quality of life. The challenge is that, because these tumors truly straddle the gastroesophageal interface, it is debated whether to apply esophageal or gastric oncological principles. It is also worth mentioning that, in the compromised patient, if the proximal margin is an issue, a transhiatal esophagectomy can also be considered.

Previous literature already suggests that not all gastroesophageal junctional tumors are equal and patients undergoing transthoracic or transhiatal approaches represent distinct patient and oncologic populations.^3^ This is why the CARDIA trial was drafted and is currently underway to compare surgical strategies for Siewert type II tumors.^4^ However, it will take a long time to mature and produce long-time data. In the meantime, retrospective analyses, such as the present study, can provide valuable interim data reflecting real-world practice, help identify factors influencing outcomes, and potentially inform ongoing and future prospective efforts.

We hypothesized that, despite differences in surgical extent and lymphadenectomy, transthoracic esophagectomy and transhiatal extended total gastrectomy are comparable in terms of oncological outcomes when appropriately selected. This study aims to add a substantial retrospective body of evidence to this knowledge gap by comparing granular oncological outcomes of transthoracic esophagectomy versus extended transhiatal gastrectomy for Siewert type II junctional cancers in our institution. Secondarily, we also included thorough descriptions of the study population and intra- and postoperative outcomes, as well as clinical versus pathologically verified lymph node patterns and recurrence patterns and long-term survival curves.

## METHODS

### Manuscript preparation

The manuscript was prepared using the Strengthening the Reporting of Observational Studies in Epidemiology (STROBE) statement.^5^

### Study design and patient selection

This retrospective observational cohort study was conducted at the University Medical Center (UMC) in Utrecht, the Netherlands. Patients were identified from a prospectively maintained institutional database including all individuals who underwent curative-intent surgery for gastroesophageal malignancies between 2013 and 2023, and all electronic patient records with tumors located at or near the gastroesophageal junction were reviewed. Initial classification was based on multidisciplinary tumor board conclusions. Tumors were classified according to the Siewert classification system based on a combination of endoscopic findings, cross-sectional imaging, and particularly metabolic activity on positron emission tomography with computed tomography (PET-CT) scans.^1^ Cases clearly defined as Siewert type I or III tumors were excluded. All cases lacking a primary Siewert classification were labeled as potential Siewert type II tumors and underwent detailed retrospective reassessment using endoscopy reports, cross-sectional imaging (computed tomography (CT) and/or PET-CT), and operative notes. This reassessment was conducted independently by three reviewers (AZ, GK, SV), including two consultant upper gastrointestinal surgeons. In cases of disagreement, consensus was achieved during a joint review meeting with two senior consultant surgeons (RvH, JR).

### Surgical procedures

Patients underwent one of the following surgical strategies based on tumor characteristics, multidisciplinary discussion, and surgeon preference:

1. Robotic or non-robotic (conventional minimally invasive or hybrid) transthoracic esophagectomy (TTE) using an Ivor Lewis approach with intrathoracic anastomosis. Anastomoses were performed using a hand-sewn end-to-side technique. At least a standard D2 + upper mediastinal (paratracheal) lymphadenectomy in the thorax was routinely performed. The anastomotic level was based on tumor location and proximal extent, and lymphadenectomy was standardized, independently of the radiation field.
2. Robotic or non-robotic (conventional minimally invasive or open) extended total gastrectomy (EG) with Roux-en-Y reconstruction. Anastomoses were performed in an end-to-side fashion using either a circular-stapled (pre-robotic and early robotic era) or a hand-sewn (more recent robotic era) technique. During an extended total gastrectomy, a transhiatal high mediastinal reconstruction is performed, requiring routine mobilization of the Roux limb, increasing the risk of tension and compromised perfusion. At least a standard D2 lymphadenectomy, as well as lymphadenectomy of the lower paraesophageal nodes, was routinely performed.
3. Robotic or non-robotic (conventional minimally invasive or hybrid) transhiatal esophagectomy with gastric conduit reconstruction and cervical anastomosis.

In addition to preoperative data, intraoperative endoscopy has recently become routine in the management of all junctional tumors and also plays a key role in guiding the surgical approach. Furthermore, in our practice, any preoperative evidence of thoracic lymph node involvement would exclude the patient from an abdominal-only approach.

To harmonize the present study with the ongoing CARDIA trial, only patients undergoing transthoracic esophagectomies or transhiatal extended total gastrectomies were included in the comparative analyses. All procedures were performed by senior consultant upper gastrointestinal surgeons RvH and JR, ensuring consistency in the quality of surgical technique as well as intraoperative decision-making.

### Neoadjuvant treatment

Neoadjuvant therapy was administered according to national guidelines and multidisciplinary tumor board recommendations. Treatment consisted of either neoadjuvant chemotherapy (different regimens over time but mostly MAGIC [Epirubicin, Cisplatin, 5-Fluorouracil], FLOT [Fluorouracil, Leucovorin, Oxaliplatin, Docetaxel] since 2019) or neoadjuvant CROSS (radiotherapy 41.4 Gy in 23 fractions with concurrent Carboplatin and Paclitaxel).

### Data collection

Baseline demographic data were collected and included age, sex, body mass index (BMI), American Society of Anesthesiologists (ASA) classification, Charlson Comorbidity Index (CCI), tumor histology, and clinical tumor and nodal staging. Intraoperative variables included surgical approach, use of robotic assistance, intraoperative endoscopy, frozen section analysis, and intraoperative changes in surgical strategy.

Pathological outcomes comprised pathological tumor and nodal stage (pT and pN), locally advanced pT-stages were collapsed into pT3–4 due to anticipated imbalance resulting from different staging criteria between esophageal and gastric cancer, resection margin status (R0/R1), proximal resection margin length, total lymph node yield, and number of positive lymph nodes. Postoperative complications were recorded and graded according to the Clavien–Dindo classification, with major complications defined as grade ≥ 3b^6^.

Recurrence sites were collected in all patients.

### Follow-up and outcomes

Patients were followed according to institutional oncological surveillance protocols. Primary outcome measures included oncological outcomes (R0-rate, proximal free margin length, total lymph node yield, positive lymph node yield). Secondary outcomes included postoperative complication rate (e.g. pneumonia and anastomotic leak), clinical versus pathology-verified lymph node patterns and sites of recurrences, as well as disease-free survival (DFS) and overall survival (OS).

### Statistics

Continuous variables were presented as medians with interquartile ranges (IQRs) or means with standard deviations, as appropriate, and compared using the Mann–Whitney *U* test.

Categorical variables were expressed as counts and percentages and compared using the chi-square or Fisher’s exact test, as appropriate.

Standard mean differences (SMDs) were calculated for all baseline variables to assess covariate balance between groups. Propensity score matching was considered if a substantial imbalance was present (SMD >0.25). Given an acceptable baseline balance and the limited sample size, multivariable regression adjustment was deemed sufficient and propensity score matching was not performed, as it would have substantially reduced statistical power without materially improving comparability.

Survival analyses were performed using the Kaplan–Meier method and compared with the log-rank test. Survival time was calculated from the date of surgery to death or last follow-up. Disease-free survival was defined as the time from surgery to the first documented recurrence or death.

Multivariate Cox proportional hazards regression analysis was used to identify factors (based on available literature) independently associated with OS, including surgical procedure, age, sex, comorbidity burden, pathological tumor stage, and major postoperative complications. Hazard ratios (HRs) with 95% confidence intervals (CIs) were reported. To reduce collinearity and overfitting in the small dataset, correlated variables were combined and the number of predictors was limited. *P*-values of <0.05 were considered statistically significant.

All statistical analyses were performed using the IBM SPSS Statistics 30.0 (Build 172) software for Mac. All statistical figures were made in Matplotlib for Python.

### Missing data

Missing data in crucial variables led to the entire data point being deleted.

Given the minor extent of missingness (<5%), imputation was unlikely to materially alter the results; thus, complete-case analysis was deemed appropriate.

## RESULTS

**Fig. 1:**
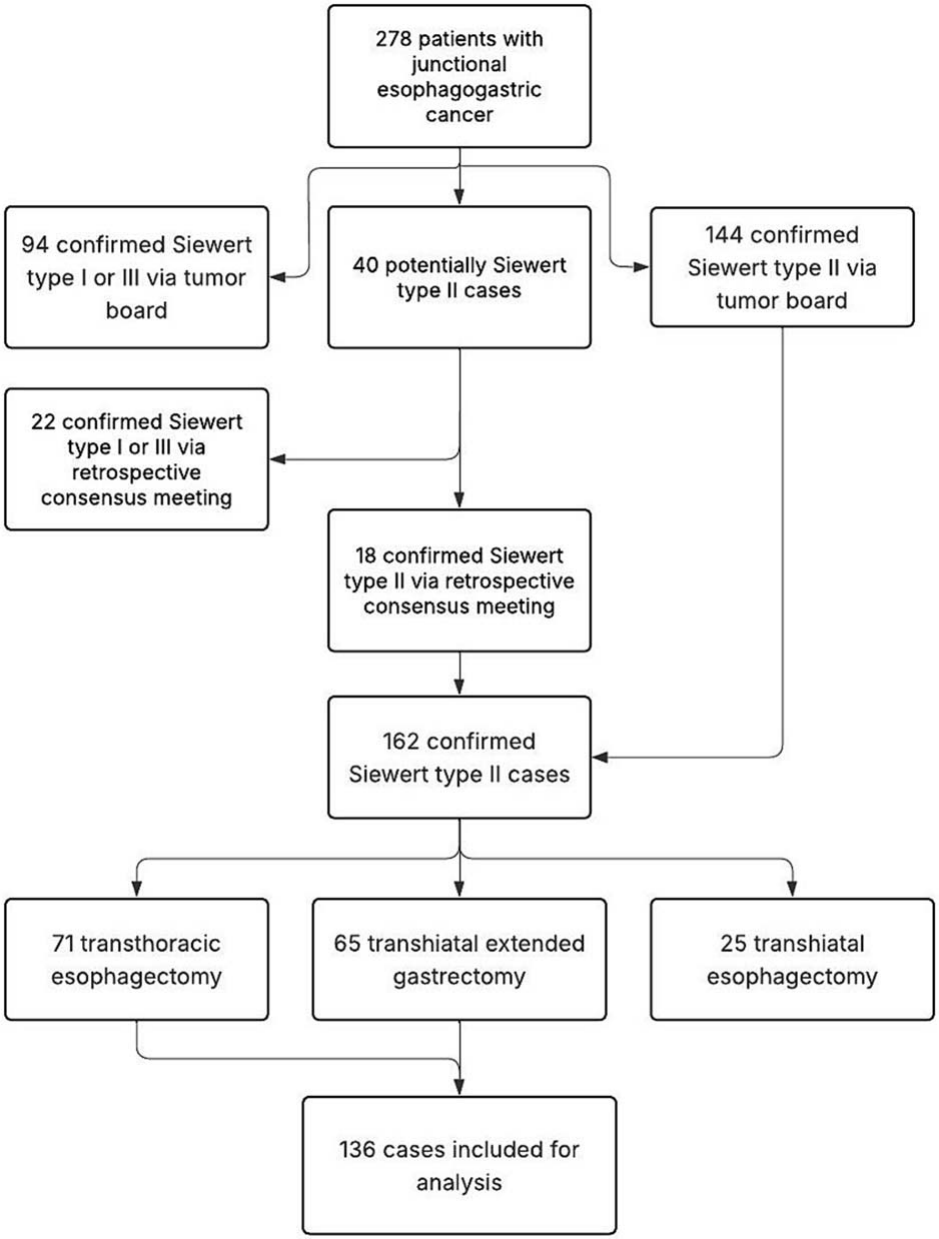
Flow chart for the patient inclusion process.

A total of 278 electronic patient records were reviewed from a prospectively maintained local database. Of these, 144 cases were confirmed as true junctional tumors (Siewert type II) based on existing multidisciplinary tumor board decisions, while an additional 40 cases were classified as potential Siewert type II tumors. Out of these 40 cases, another 18 cases were confirmed as Siewert type II based on the described expert consensus review. Out of the 162 patients comprising the study cohort, 71 underwent transthoracic esophagectomy (Ivor Lewis procedure), 65 underwent transhiatal extended total gastrectomy with Roux-en-Y reconstruction, and 25 underwent transhiatal esophagectomy with gastric conduit reconstruction and cervical anastomosis. The patient inclusion process is illustrated in Figure 1.

Table 1 summarizes baseline characteristics. Median age, BMI, ASA class ≥3, comorbidity burden (CCI), clinical T stage, nodal status, and histological subtype did not differ significantly. A higher proportion of males was observed in the transthoracic esophagectomy group (91.5% vs. 70.8%, *P* = 0.004).

**Table 1:** Baseline data

	TTE (*n* = 71)	EG (*n* = 65)	*P*-value/SMD
Age, median [IQR]	66 [59–72]	65 [58–71]	0.628/0.093
Gender (M), *n* (%)	65 (91.5)	46 (70.8)	0.004/0.531
BMI, median [IQR]	26.2 [24.2–29]	25.6 [23.3–28.6]	0.532/0.095
ASA ≥ 3, *n* (%)	24 (33.8)	21 (32.3)	0.998/0.149
CCI, median [IQR]	6 [6–7]	6 [6–7]	0.687/0.032
Neoadjuvant treatment Chemotherapy, *n* (%) Chemoradiotherapy, *n* (%)	68 (95.8) 26 (36.6) 42 (59.2)	59 (90.8) 55 (84.6) 4 (6.2)	0.408/0.200 <0.001 <0.001
cT3–4	51 (71.8)	47 (72.3)	1.000/0.011
cN+	32 (45.1)	25 (38.5)	0.207/0.246

Most patients received neoadjuvant treatment, with no significant difference in overall use between groups. However, treatment modality differed substantially: chemotherapy alone was more common in the extended gastrectomy versus the transthoracic esophagectomy group (84.6% [*n* = 55] vs. 36.6% [*n* = 22], *P* < 0.001), whereas chemoradiotherapy predominated in the transthoracic esophagectomy group (59.2% [*n* = 42] vs. 6.2% [*n* = 4], *P* < 0.001).

SMDs for all baseline characteristics were between <0.10 and < 0.20, except for cN+ (0.246) and gender (0.531).

Intraoperative characteristics are summarized in Table 2. Transthoracic esophagectomy was more frequently performed using a robotic approach (94.4% [*n* = 67] vs. 41.5% [*n* = 27], *P* < 0.001). The use of intraoperative endoscopy was similar in groups (74.3% [*n* = 52] vs. 66.2% [*n* = 43], *P* = 0.398). In contrast, intraoperative frozen section analysis was performed significantly more often during extended gastrectomy (90.8% [*n* = 59] vs. 4.2% [*n* = 3], *P* < 0.001). Intraoperative strategy was altered in eight cases (6%), of which six patients initially planned for extended total gastrectomy ultimately underwent transthoracic esophagectomy instead.

**Table 2:** Intraoperative data

	TTE (*n* = 71)	EG (*n* = 65)	*P*-value
Robotic, *n* (%)	67 (94.4)	27 (41.5)	<0.001
Intraoperative endoscopy, *n* (%)	52 (74.3)	43 (66.2)	0.398
Intraoperative frozen section, *n* (%)	3 (4.2)	59 (90.8)	<0.001
Intraoperative policy change, *n*	6 patients who were initially planned for extended gastrectomy eventually underwent transhiatal esophagectomy. 2 patients who were initially planned for extended gastrectomy eventually underwent transthoracic esophagectomy.		

Pathological outcomes are summarized in Table 3. Pathological T stage differed significantly between groups (*P* < 0.001). Patients undergoing extended total gastrectomy more frequently had pT4 disease (35.4% [*n* = 23] vs. 5.6% [*n* = 4]), whereas pT3 tumors were more common after transthoracic esophagectomy (45.1% [*n* = 32]) vs. the extended total gastrectomy group. Pathological nodal stage distribution was similar in groups (*P* = 0.786), and the proportion of R0 resections was comparable for esophagectomy (94.4%, *n* = 67) vs. gastrectomy (86.0% [*n* = 56], *P* = 0.217). Proximal resection margins were significantly longer following transthoracic esophagectomy (*P* < 0.001). Margins ≥50 mm were achieved in 77.4% (*n* = 55) of esophagectomy cases compared with 1.5% (*n* = 1) after extended gastrectomy, whereas margins <10 mm occurred exclusively in the gastrectomy group. Median total lymph node yield was higher after transthoracic esophagectomy (45 [IQR 33.5–51.5] vs. 28 [IQR 19–39], *P* < 0.001), while the number of positive lymph nodes did not differ significantly between groups (median 1 [IQR 0–2] for transthoracic esophagectomy vs. median 1 [IQR 0–4] for extended total gastrectomy).

**Table 3:** Pathology data

	TTE (*n* = 71)	EG (*n* = 65)	*P*-value
Histology, *n* (%) Intestinal Diffuse Mixed Unknown	39 (54.9) 7 (9.9) 2 (2.8) 23 (32.4)	35 (53.8) 9 (13.8) 2 (3.1) 19 (29.2)	0.900
pT, *n* (%) Tx T1 T2 T3 T4	17 (23.9) 10 (14.1) 8 (11.3) 32 (45.1) 4 (5.6)	7 (10.8) 10 (15.4) 3 (4.6) 22 (33.8) 23 (35.4)	<0.001
pN, *n* (%) N0 N1 N2 N3	33 (46.5) 16 (22.5) 14 (19.7) 8 (11.3)	25 (38.5) 16 (24.6) 14 (21.5) 10 (15.4)	0.786
R0, *n* (%)	67 (94.4)	56 (86)	0.217
Proximal margin, mm <1 mm, *n* (%) 1–5 mm, *n* (%) 6–10 mm, *n* (%) 10–50 mm, *n* (%) 50–100 mm, *n* (%) >100 mm, *n* (%) Unknown, *n* (%)	0 (0) 1 (1.4) 0 (0) 11 (15.5) 39 (54.9) 16 (22.5) 4 (5.6)	8 (12.3) 22 (33.8) 18 (27.7) 10 (15.4) 0 1 (1.5) 6 (9.2)	<0.001
Lymph node yield total, median [IQR]	45 [33.5–51.5]	28 [19–39]	<0.001
Lymph node yield, positive, median [IQR]	1 [0–2]	1 [0–4]	0.286

Postoperative complications are presented in Table 4. The rate of major complications (Clavien–Dindo ≥3b) was comparable between transthoracic esophagectomy and extended gastrectomy (38.0% [*n* = 27] vs. 38.5% [*n* = 25], *P* = 1.000). No significant differences were observed in anastomotic leaks (21.1% [*n* = 15] vs. 30.8% [*n* = 20], *P* = 0.276) or postoperative pneumonia (23.9% [*n* = 17] vs. 21.5% [*n* = 14], *P* = 0.897).

**Table 4:** Complications data

	TTE (*n* = 71)	EG (*n* = 65)	*P*-value
Overall Clavien ≥3b, *n* (%)	27 (38)	25 (38.5)	1.000
Anastomotic leak, *n* (%)	15 (21.1)	20 (30.8)	0.276
Pneumonia, *n* (%)	17 (23.9)	14 (21.5)	0.897

**Fig. 2:**
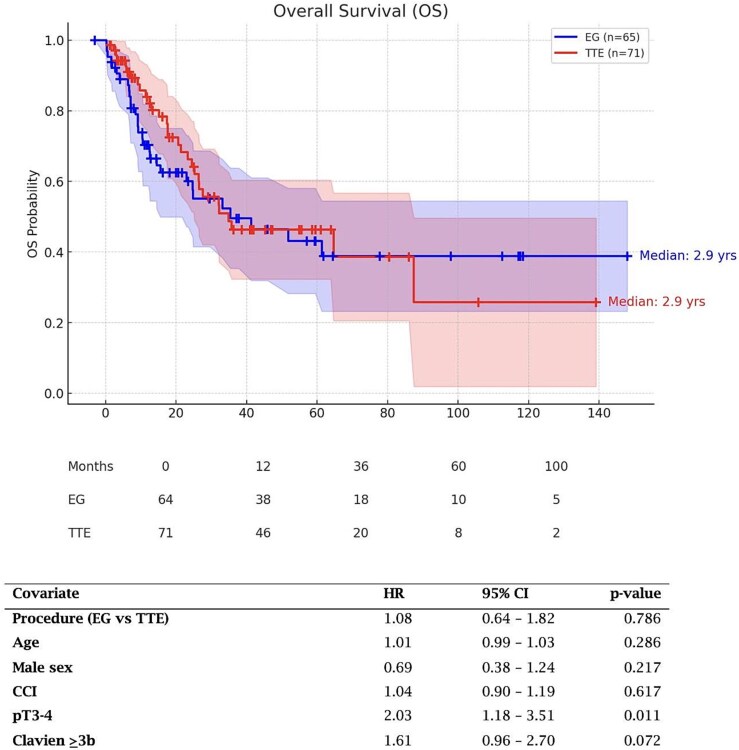
Overall survival. Kaplan–Meier analysis demonstrated no significant difference (log-rank *P* = 0.611) in overall survival between patients undergoing transthoracic esophagectomy vs extended total gastrectomy. In multivariate Cox regression analysis, surgical procedure was not associated with overall survival (aHR 1.08, 95% CI 0.64–1.82; *P* = 0.786). Advanced pathological tumor stage (pT3–4) was independently associated with worse overall survival (aHR 2.03, 95% CI 1.18–3.51; *P* = 0.011), whereas age, sex, comorbidity burden, and major postoperative complications were not.

Overall survival did not differ significantly between patients undergoing extended gastrectomy and transthoracic esophagectomy (Figure 2). Median follow-up time was 18 months. Median OS was 2.9 years in both groups, and Kaplan–Meier analysis demonstrated no significant difference (log-rank *P* = 0.611).

In multivariate Cox regression analysis, surgical procedure was not associated with OS (aHR 1.08, 95% CI 0.64–1.82; *P* = 0.786). Advanced pathological tumor stage (pT3–4) was independently associated with worse OS (aHR 2.03, 95% CI 1.18–3.51; *P* = 0.011), whereas age, sex, comorbidity burden, and major postoperative complications were not.

**Fig. 3:**
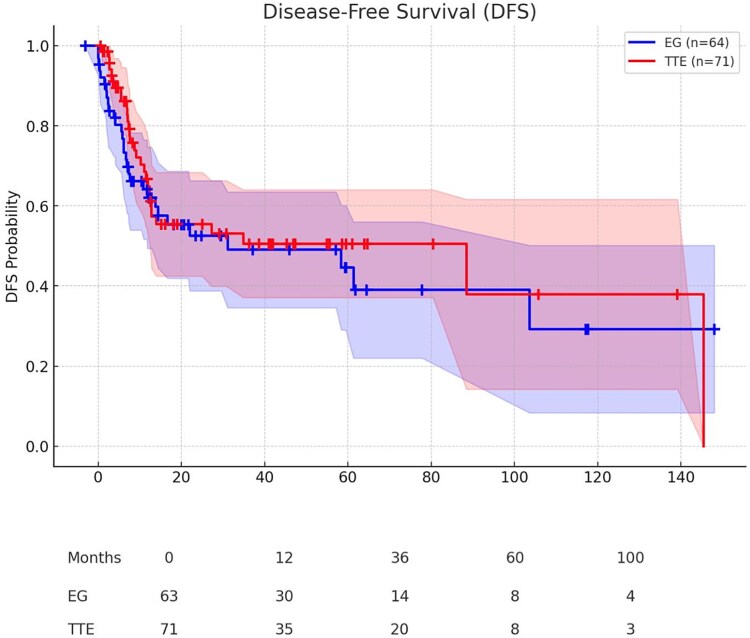
Disease-free survival. Disease-free survival was also comparable between groups. Kaplan–Meier analysis showed no significant difference in DFS between extended total gastrectomy and transthoracic esophagectomy (log-rank *P* = 0.530).

DFS was also comparable between groups (Figure 3). Kaplan–Meier analysis showed no significant difference in DFS between extended total gastrectomy and transthoracic esophagectomy (log-rank *P* = 0.530).

**Fig. 4:**
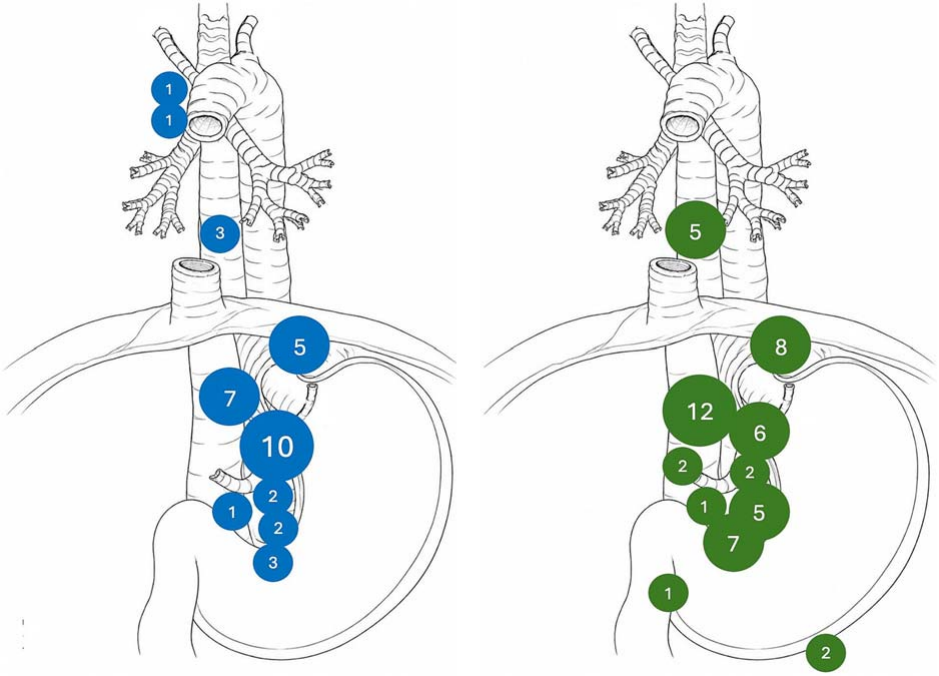
Pathologically verified lymph node patterns. The number in the circle represents the number of patients with pathologically confirmed lymph node metastasis at the indicated location. The circle size represents the number of patients. Transthoracic esophagectomy (TTE) to the left and extended total gastrectomy (EG) to the right.

Clinical and pathological lymph node involvement patterns are illustrated in Figure 4 and show a significant overlap in abdominal stations 8, 1, 2, and 7.

**Fig. 5:**
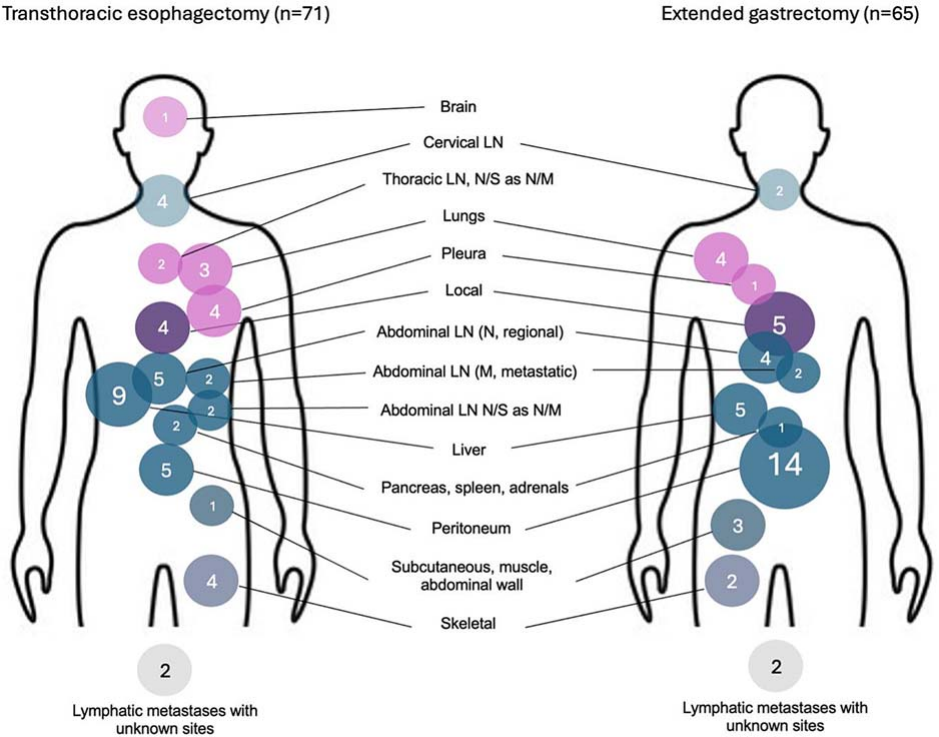
Recurrence site patterns. Patterns of recurrence sites. Numbers indicate the frequency at each site. Larger circles represent a higher number of recurrences. Transthoracic esophagectomy to the left and extended total gastrectomy to the left.

Recurrence site patterns are illustrated in Figure 5. Overall, recurrence occurred across a broad range of anatomical sites in both surgical groups. Lymphatic and peritoneal recurrences constituted the most frequent patterns following both transthoracic esophagectomy and extended gastrectomy. Distant metastatic spread to solid organs, including the liver, lungs, and peritoneum, was observed in both groups.

## DISCUSSION

This study on patients with true gastroesophageal junction (Siewert type II) tumors showed clear differences in lymph node yield and proximal resection margin lengths between approaches. However, interestingly, this did not translate into significant differences in OS and DFS between groups.

One of the most pronounced differences between the two procedures was the significantly higher lymph node yield after transthoracic esophagectomy, reflecting the more extensive mediastinal lymphadenectomy inherent to this approach. Nevertheless, pathological nodal stage distribution and the number of positive lymph nodes did not differ between groups. Importantly, previous large population-based analyses and meta-analyses have demonstrated that a higher lymph node yield is independently associated with improved OS, even in patients treated with neoadjuvant chemoradiotherapy.^7,8^ These findings suggest that an extended lymphadenectomy may provide therapeutic benefit beyond accurate staging alone, potentially through improved regional disease control. In the present cohort of Siewert type II tumors, however, comparable nodal stage distribution and survival outcomes between groups may indicate that, when preoperative staging and intraoperative assessment are carefully performed, a tailored lymphadenectomy strategy can achieve oncologic adequacy without necessarily requiring maximal mediastinal dissection in all cases. The low incidence of positive mediastinal lymph nodes in the transthoracic esophagectomy group likely reflects the predominantly abdominal lymphatic drainage of Siewert type II tumors (Figure 4), with relatively infrequent involvement of the upper mediastinum. Although neoadjuvant radiotherapy may have contributed to nodal downstaging, 40% of patients did not receive radiotherapy, suggesting this finding is not solely treatment-related.

A significantly higher proportion of T4 tumors was seen in the extended total gastrectomy group, likely mainly due to differences in TNM classification. Gastric cancers are classified as T4 with serosal penetration, whereas esophageal tumors require invasion of adjacent organs to be considered T4, to some extent artificially inflating T4 in the gastrectomy group.^9^ Proximal resection margins were substantially longer following transthoracic esophagectomy, whereas shorter or positive margins were frequently observed after extended gastrectomy. Importantly, however, R0 resection rates were comparable between both groups, and margin length did not appear to influence survival outcomes. This suggests that achieving a microscopically radical resection, as defined by the College of American Pathologists, is more relevant than absolute margin length, particularly in tumors located at the gastroesophageal junction where anatomical constraints often dictate surgical strategy, which is also supported by previous literature.^10^ Postoperative morbidity occurred in one-third of the patients in both groups and did not differ significantly. Notably, the comparable rates of postoperative pneumonia and major complications were somewhat unexpected, questioning the traditional arguments in favor of extended gastrectomy as being the least invasive and most tolerable procedure with fewer pulmonary complications, challenging the commonly held assumption of higher postoperative morbidity in two-field surgery. This should, however, be interpreted in context, as more than half of the EGs were performed non-robotically, whereas nearly 95% of procedures in the TTE group were robotic, potentially introducing bias in the observed morbidity of the EG group. The discrepancy is mostly attributable to the later adoption of robotic surgery for EG.^11^

The lack of difference in oncological outcomes between the two approaches is likely multifactorial. Importantly, it may reflect high-quality, individualized clinical decision-making in daily practice, particularly within multidisciplinary tumor board discussions. Careful patient selection, nuanced interpretation of tumor extent, and tailored choice of surgical strategy may allow both procedures to achieve equivalent oncological results when applied appropriately. In this context, the observed equivalence should not be interpreted as the absence of meaningful differences between procedures but rather as an indication that each approach can be oncologically sound when chosen for the right patient. Patterns of lymphatic involvement and recurrence further support this interpretation. Substantial overlap was observed in involved lymph node stations and recurrence sites between groups, highlighting the biological heterogeneity of Siewert type II tumors and the difficulty of strictly categorizing them as either esophageal or gastric cancers. Nevertheless, the overlap in—especially thoracic—recurrence sites is surprising, giving the radical thoracic lymphadenectomy and resection in the TTE group compared to a minimal thoracic invasion in the EG group. However, thoracic recurrences were mostly local (anastomosis, pleura) or hematogenous (lungs). Another explanation for this overlap could be the relocalization of non-resected abdominal lymph nodes along the greater curvature, which might have spread disease. Additionally, the broad distribution of recurrence sites suggests that tumor biology, rather than surgical approach alone, plays a dominant role in long-term outcomes.^12,13^ Also, there were no recurrences in mediastinal or subcarinal nodes in the extended gastrectomy group, suggesting that these tumors truly behaved more like gastric cancers. In this context, our findings complement existing literature and are particularly relevant until prospective randomized data become available.

Existing literature on this topic remains heterogeneous, with conflicting results depending on study design, patient selection, and endpoints. Population-based data from the Dutch Upper Gastrointestinal Cancer Audit demonstrated largely comparable long-term survival, postoperative morbidity, and pathological outcomes between esophagectomy and gastrectomy for gastroesophageal junction tumors, supporting the notion that both procedures can be oncologically adequate when appropriately applied.^14^ In contrast, a recent multinational high-volume center cohort analysis reported a survival advantage for transthoracic esophagectomy, particularly after propensity score matching and in selected subgroups, attributing this effect to higher R0 resection rates and lymph node yields.^15^ Earlier studies and systematic reviews further illustrate the lack of consensus. Several meta-analyses have reported no clear survival difference between transhiatal and transthoracic approaches, despite differences in lymphadenectomy extent and postoperative morbidity profiles.^2,16^ Registry-based analyses and institutional series have variably favored esophagectomy or gastrectomy, while others suggested equivalence, highlighting the influence of selection bias and institutional practice patterns.^3,17–22^ Importantly, long-term quality-of-life data suggest that although functional outcomes differ between procedures, overall quality of life may be largely comparable, reinforcing the need to consider patient-centered outcomes alongside oncological endpoints.^23,24^

Several limitations must be acknowledged. Although baseline characteristics were largely comparable, the retrospective design introduces inherent risks of selection bias and residual confounding, including bias by indication, as the choice of procedure—as well as the type of neoadjuvant treatment (chemoradiotherapy vs. chemotherapy alone)—was guided by tumor characteristics and multidisciplinary clinical judgment. However, this ought to reflect real-world multidisciplinary decision-making rather than random allocation. Additionally, apart from the recently published ESOPEC data—which were not available during the inclusion period—there has been no conclusive evidence demonstrating the superiority of any of the two neoadjuvant strategies, and its findings remain subject to ongoing debate.^25–27^ Moreover, by using a prospectively maintained database and including all consecutive patients, selection and reporting bias were minimized to the best of our possibilities. Furthermore, this was a single-center study performed by highly experienced surgeons, which may limit generalizability to lower-volume settings—on the other hand, ensuring consistency in the quality of surgical technique, as well as intraoperative decision-making. Quality-of-life outcomes, which may differ between surgical approaches, were not assessed and warrant further investigation, which will be addressed in the CARDIA trial.^4^

In conclusion, both transthoracic esophagectomy and extended transhiatal gastrectomy provide comparable oncological outcomes and postoperative morbidity in patients with Siewert type II gastroesophageal junction tumors. These results support an individualized, multidisciplinary approach to surgical decision-making, emphasizing patient- and tumor-specific factors, as well as institutional expertise influencing outcomes, rather than adherence to a single standardized surgical strategy.

## Data Availability

All data are available upon reasonable request.

## Notes

Specific author contributions: AZ and GK proposed the research idea. All authors contributed to design, protocol, and planning of the study. AZ and GK performed the literature search and wrote the first manuscript draft. Data collection and analyses were performed by AZ, GK, and SV. All authors contributed to several rounds of revisions. All authors read and approved the final manuscript for submission.

Financial support: The authors received no specific funding for this work.
